# An application of Extended Normalisation Process Theory in a randomised controlled trial of a complex social intervention: Process evaluation of the Strengthening Families Programme (10–14) in Wales, UK

**DOI:** 10.1016/j.ssmph.2017.01.002

**Published:** 2017-01-11

**Authors:** Jeremy Segrott, Simon Murphy, Heather Rothwell, Jonathan Scourfield, David Foxcroft, David Gillespie, Jo Holliday, Kerenza Hood, Claire Hurlow, Sarah Morgan-Trimmer, Ceri Phillips, Hayley Reed, Zoe Roberts, Laurence Moore

**Affiliations:** a Centre for the Development and Evaluation of Complex Interventions for Public Health Improvement (DECIPHer), Cardiff School of Social Sciences, Cardiff University, 1-3 Museum Place, Cardiff CF10 3BD, United Kingdom; b Cardiff School of Social Sciences, Cardiff University, Glamorgan Building, King Edward VII Avenue, Cardiff CF10 3WT, United Kingdom; c Faculty of Health and Life Sciences, Oxford Brookes University, Marston Campus, Oxford OX3 0FL, United Kingdom; d South East Wales Trials Unit, Centre for Trials Research, Cardiff University, Heath Park, Cardiff CF14 4YS, United Kingdom; e Swansea Centre for Health Economics, College of Human and Health Sciences, Swansea University, Singleton Park, Swansea SA2 8PP, United Kingdom; f Centre for Medical Education, School of Medicine, Cardiff University, Heath Park, Cardiff CF14 4XN, United Kingdom; g MRC/CSO Social and Public Health Sciences Unit, University of Glasgow, Top floor, 200 Renfield Street, Glasgow G2 3QB, United Kingdom

**Keywords:** United Kingdom, Strengthening Families Programme 10–14, Family-based prevention, Randomised controlled trial, Process evaluation, Implementation, Fidelity, Extended Normalisation Process Theory

## Abstract

**Purpose:**

Process evaluations generate important data on the extent to which interventions are delivered as intended. However, the tendency to focus only on assessment of pre-specified structural aspects of fidelity has been criticised for paying insufficient attention to implementation processes and how intervention-context interactions influence programme delivery. This paper reports findings from a process evaluation nested within a randomised controlled trial of the Strengthening Families Programme 10–14 (SFP 10–14) in Wales, UK. It uses Extended Normalisation Process Theory to theorise how interaction between SFP 10–14 and local delivery systems - particularly practitioner commitment/capability and organisational capacity - influenced delivery of intended programme activities: fidelity (adherence to SFP 10–14 content and implementation requirements); dose delivered; dose received (participant engagement); participant recruitment and reach (intervention attendance).

**Methods:**

A mixed methods design was utilised. Fidelity assessment sheets (completed by practitioners), structured observation by researchers, and routine data were used to assess: adherence to programme content; staffing numbers and consistency; recruitment/retention; and group size and composition. Interviews with practitioners explored implementation processes and context.

**Results:**

Adherence to programme content was high - with some variation, linked to practitioner commitment to, and understanding of, the intervention’s content and mechanisms. Variation in adherence rates was associated with the extent to which multi-agency delivery team planning meetings were held. Recruitment challenges meant that targets for group size/composition were not always met, but did not affect adherence levels or family engagement. Targets for staffing numbers and consistency were achieved, though capacity within multi-agency networks reduced over time.

**Conclusions:**

Extended Normalisation Process Theory provided a useful framework for assessing implementation and explaining variation by examining intervention-context interactions. Findings highlight the need for process evaluations to consider both the structural and process components of implementation to explain whether programme activities are delivered as intended and why.

## Highlights

• Uses Extended Normalisation Process Theory to theorise intervention implementation.
• The Strengthening Families Programme was delivered with good overall fidelity.
• Adherence to planned content and staffing guidelines was high, with some variation.
• Recruitment of families was challenging and affected group size/composition
• Intervention-context interactions shaped variation in fidelity and implementation.

## 1 Introduction

Adolescent substance misuse is a significant problem in developed countries (Currie et al., 2012; U.S. Department of Health and Human Services, 2007) and early initiation of substance use is associated with higher levels of substance-related harm during adulthood (Dawson et al., 2008, Grant and Dawson, 1998). Because the consequences of early initiation are difficult to modify, an important response has been the development of family-based prevention interventions (Cuijpers, 2003, Kumpfer et al., 2003). One such intervention, the Strengthening Families Programme (SFP), aims to delay substance use initiation and prevent later misuse through strengthening family-based protective factors. In the United States of America trials of SFP 10-14 - a universal version of SFP for families with children aged 10–14, have found evidence of long-term effectiveness (Spoth, Redmond & Shin, 2001; Spoth et al., 2013; Spoth, Redmond, Trudeau & Shin, 2002), though the methodological rigour of these studies has been criticised (Gorman, 2015). The evidence base for family-based prevention interventions such as SFP 10-14 is dominated by studies from the USA and there is a need for more research on whether effective interventions can be successfully ‘transported’ to other national contexts (Petrie, Bunn & Byrne, 2007), where they are more likely to be implemented under ‘real-world’ conditions, and without extensive input from programme developers (Axford & Morpeth, 2013).

Family-based programmes are complex interventions, with multiple components designed to work synergistically. Process evaluations, which analyse implementation, aid interpretation of complex outcome effects and understanding of intervention theory (Durlak, 1998, Durlak and DuPre, 2008). An important purpose of process evaluations is to assess the extent to which interventions are implemented with fidelity (Carroll et al., 2007, Moore et al., 2014). This includes adherence (whether planned activities are delivered), dose (how much of an intervention is delivered/received), delivery quality, and reach and recruitment (Baranowski and Stables, 2000, Dusenbury et al., 2003). Alongside these quantitative measures, qualitative research can provide important data on the processes which influence implementation, and their variation across contexts (Moore et al., 2014).

New interventions must operate within existing delivery systems and they depend upon cooperation from individuals and organisations, especially when delivered on a multi-agency basis (May, 2013) - a common social service delivery mechanism in the UK and elsewhere. Delivery settings are typically complex systems - characterised by the interaction of multiple individuals, social networks and organisations. Within these systems practitioners make meaning of interventions in ways which shape how they are delivered (Bisset et al., 2009, May, 2013) – though the study of these phenomena is limited (Bisset et al., 2013, Hill et al., 2007). Practitioner engagement with an intervention may be emergent (and therefore hard to predict), and self-adaptive rather than centrally controlled (Sterman, 2006; Tan, Wen & Awad, 2005). Although studies in many countries have encountered variation in implementation across delivery contexts (Cantu et al., 2010, Durlak and DuPre, 2008, Lendrum and Humphrey, 2012), the role of intervention-context interaction in shaping this has often been overlooked (Bisset et al., 2009; Bonell, Fletcher, Morton, Lorenc & Moore, 2012; Glasgow, Lichtenstein & Marcus, 2006; Hawe, Shiell, Riley & Gold, 2004), and the narrow focus of process evaluations on quantitative assessment of pre-specified structural aspects of interventions (e.g. coverage of intervention activities) has been criticised for paying insufficient attention to the processes through which they occur (Bisset et al., 2009, Hawe et al., 2004). A previous trial of SFP 10-14 (conducted in the United States) – in which the programme was delivered by community-university partnerships, found no significant association between implementation team functioning and levels of adherence, but suggested that potential relationships may have been masked by the consistently high rates of adherence across programmes (Spoth, Guyll, Lillehoj & Redmond, 2007). However, evaluation of the programme in the USA as part of ‘real world’ dissemination found greater variation in adherence and other aspects of implementation (staffing levels, group size, children's age range), though no clear association between facilitator characteristics and fidelity (Cantu et al., 2010, Hill et al., 2007). Questions therefore remain about the key influences on the quality of implementation of SFP 10-14, the role of individual facilitators and their teams, and the influence of wider contextual factors.

Increasing attention is therefore being paid to intervention-context interactions, and their influence on implementation processes and hypothesized outcomes (Moore et al., 2014). It is important to understand how practitioners engage with interventions because this can provide insights into why fidelity and intervention effectiveness vary over space and time, and the extent to which an intervention may be adopted. One important contribution to the study of these processes is Extended Normalisation Process Theory (ENPT) (May, 2013) which seeks “to provide a more comprehensive explanation of the constituents of implementation processes” by integrating existing theories that are more concerned with specific processes, such as intervention delivery, integration and normalisation. ENPT conceptualises implementation as comprising practitioners - who have agency that is manifested when they interact with each other and with intervention components; and implementation contexts comprising “the socio-structural and social-cognitive resources that people draw on to realise that agency”. It therefore offers a useful framework for explaining implementation processes and the role played by intervention-context interactions.

ENPT has four main constructs. First, *potential* concerns practitioners’ commitment to deliver an intervention and behave in ways which are congruent with its aims, underpinning the action necessary to embed it within agents’ working practice (May, 2013). Whether practitioners value the changes an intervention brings about (change valence) and perceive that the changes are feasible within their local context (change efficacy), determine levels of commitment (Weiner, 2009). Second, *capability* concerns the possibilities presented by the intervention. *Capability* comprises: workability - how practitioners adjust what they do when organising an intervention - for example, (re)allocation of roles and responsibilities; and integration - how practitioners perceive implementation of an intervention to be linked to the wider social system. Third, *capacity* is the structure into which an intervention is introduced. Implementation depends on agents’ co-operation to accommodate the intervention by modifying norms and roles in social systems and redistributing resources, e.g. providing funding (May, 2013).

*Potential*, *capability* and *capacity* form the context for the fourth construct - *contribution*. This comprises the ways in which practitioners make sense of a complex intervention and their role in delivering it, the enactment of the intervention itself, and reflexive monitoring of its effects. The enactment of the intervention is expected to have specific qualities for each process evaluation component (e.g. adherence to programme manuals). Differences and similarities between expectations and practice can thus be explained in terms of *potential*, *capability* and *capacity*.

A small number of empirical studies have employed ENPT as a theoretical framework to understand the implementation of interventions within healthcare systems (the REFReSH study group, 2015, Thomas et al., 2015). Our paper – which applies ENPT to a social intervention outside the healthcare system, reports findings from a process evaluation within a randomised controlled trial of the Strengthening Families Programme (SFP) 10-14UK. We build on previous studies which have employed ENPT mainly to analyse implementation processes, by extending its application to explain how such processes shape the extent to which programme inputs and activities occur as intended: fidelity (adherence to SFP 10–14 content and implementation requirements); dose delivered (number of programmes organised); dose received (participant interest/engagement); reach (the proportion of families in the intervention group that participated in SFP 10–14); and participant recruitment (Linnan & Steckler, 2002). In doing so, this paper aids interpretation of outcome effects within the trial, develops our understanding of how SFP 10–14 could be implemented in a UK setting, and assesses the potential for ENPT to contribute to understanding of implementing social interventions within complex systems. The paper also extends previous process evaluations of SFP 10-14 which have focused mainly on the quantitative assessment of implementation (e.g. Byrne, Miller, Aalborg, Plasencia & Keagy, 2010; Spoth et al., 2007; Spoth, Guyll, Trudeau & Goldberg-Lillehoj, 2002).

## 2 Methods

### 2.1 Study design

The study was a process evaluation embedded within Project SFP Cymru – a pragmatic cluster randomised controlled trial of SFP 10-14 UK (with families as the unit of randomisation), conducted in seven counties of Wales. The study protocol was approved by The Research Ethics Committee for Wales (reference 09/MRE09/53). Full details are given in Segrott et al. (2014).

### 2.2 Structures and systems for programme delivery

County councils and their partner agencies with responsibility for parenting support/substance misuse prevention were invited to apply to the Welsh Government for funding to implement SFP 10-14 as part of the randomised controlled trial (none of these counties had previously implemented the programme). This process was designed to maximise the external validity of the trial by basing implementation on the systems and agencies which would be likely to deliver SFP 10-14 beyond the life of the RCT. Six county level partnerships were selected to implement the programme (one additional county later joined the study). Three counties were funded by Welsh Government, and three by the trial grant. Levels of funding provided (circa £75k per annum per county) were based on prior experience of running SFP 10-14 outside a research context. Funding covered the costs of employing coordinators and administrators, facilitator training, and programme delivery (e.g. room hire, participant transportation). Programme manuals and materials (e.g. activity sheets and DVDs) were provided to each team free of charge (and could continue to be used after the end of the trial). The local agency partnership in each county selected a lead organisation which appointed a co-ordinator who identified families eligible to participate in the trial, and organised and facilitated SFP 10–14 programmes. Coordinators’ managers normally held senior leadership roles within their organisation, with responsibilities for securing ongoing funding for programmes, and managing the broader services within which SFP 10-14 sat.

Practitioners from local agencies were trained free of charge as SFP 10–14 facilitators in return for a commitment by their employer that the individuals concerned would be available to facilitate SFP 10–14 during all seven weeks of at least one programme, with the intention that programmes would be delivered by multi-agency teams. Two trainers provided 3½-day training courses in all counties. A second round of courses was subsequently held which included training for two facilitators in each county to become trainers, so that delivery teams could maintain their staffing levels over time. Programmes were delivered in schools and community facilities between 2010 and 2012, with a target group size of 10–12 families.

### 2.3 Recruitment and randomisation

Promotion of the trial in the community generated self-referrals from families, and practitioners working in local agencies could refer families. Access to SFP 10-14 was only available to families who agreed to participate in the trial and who were allocated to the intervention arm. Programme coordinators visited families who were referred or self-referred to the trial to provide information about the intervention and confirm eligibility. Based on this visit, and information contained in referral/self-referral forms, coordinators determined whether a family was likely to experience/present challenges within a group setting. Eligible families who agreed to be contacted by the research team were visited by a researcher who sought consent for participation in the trial, and collected baseline data from consenting parents/carers and children. Following baseline data collection families were randomised on a 1:1 ratio to receive SFP 10-14 alongside existing services (intervention arm) or continue receiving existing services only (control arm). The study had a target sample size of 756 families.

### 2.4 SFP 10-14 UK intervention

The seven-week SFP 10-14 universal prevention intervention is delivered to groups of families (Molgaard, Spoth & Redmond, 2000). Participants divide into separate groups of parents/carers and children during the first hour of weekly sessions and meet for a refreshment break followed by the final hour when parents/carers and children come together in family groups. Facilitators use videos, interactive teaching and games specified in the programme manual to demonstrate and support the practice of parenting and other skills. The UK version of SFP 10–14 uses videos featuring actors with UK accents and incorporates changes to makes activities more appropriate for a UK context (Allen, Coombes & Foxcroft, 2007). Otherwise it closely follows the American version.

**Fig. 1:**
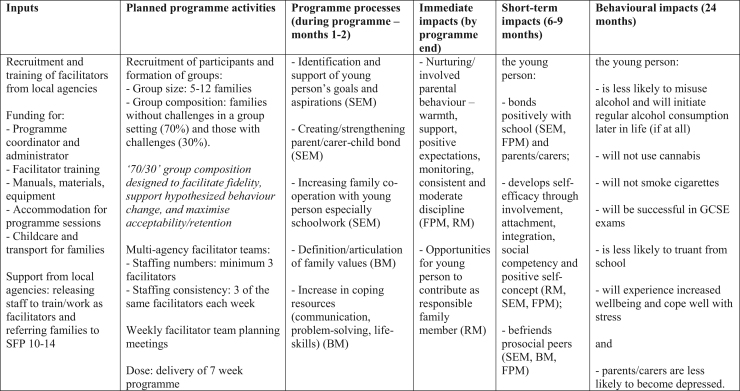
: Logic model for SFP 10–14. Underpinning theories: BM – biopsychosocial model (Kumpfer, Trunnell & Whiteside, 1990); FPM – family process model (Conger et al., 1992, Conger et al., 1993); RM – resiliency model (Richardson, Neiger, Jensen & Kumpfer, 1990); SEM – social ecology model (Kumpfer & Turner, 1990).

Fig. 1 shows the intervention logic model - developed by the paper authors, and reviewed by one of the programme developers. Project SFP Cymru implementation guidelines included provision of free transport, childcare and refreshments for families; and participant groups composed of approximately 30 percent of families who might present or experience challenges within a group setting (“families with challenges”) and 70 percent who would not (“families without challenges”) (Segrott, 2013). This was a response to the difficulties of delivering the programme mainly to families with challenging behaviour, and had three aims. Firstly to assist implementation fidelity, by reducing disruptions to programme activities. Secondly to optimise hypothesized behaviour change processes, through creating pro-social group dynamics. Thirdly to maximise participant retention by achieving a participant-staff ratio that allowed support to be provided for families with higher needs/challenges, and create an enjoyable learning environment. This group composition strategy facilitated a proportionate universalism approach (Marmot et al., 2010), whereby participation was invited on a universal basis while recognising that greater efforts were needed to involve certain groups.

### 2.5 Methods and data collection

Table 1 provides an overview of quantitative data sources used to assess implementation. Based on the framework developed by Linnan and Steckler (2002), these encompassed fidelity, dose delivered and received, reach, and programme inputs. Semi-structured interviews with programme staff evaluated intervention-context interactions and their variation across sites.

**Table 1:** Quantitative data sources used to assess implementation of SFP 10–14 and process evaluation components (Linnan & Steckler, 2002).

**Process evaluation component**	**Programme component**	**Data source**	**Indicator**
Dose delivered		Co-ordinator data	Number of programmes (and constituent sessions) delivered
			
Fidelity	SFP 10–14 manual - guidance on content (adherence)	Fidelity assessment sheets, observer scores	Percentage of activities reported as completely/mostly covered
		
	Training guidance on staffing	Co-ordinator data	Percentage of programmes with ≥3 facilitators at every session
		Co-ordinator data	Percentage of programmes with ≥3 of the same facilitators at every session
	Guidance on group size	Co-ordinator data	Percentage of programmes with more than four^a^ and fewer than thirteen families
	SFP 10-14 UK approach - group composition	Co-ordinator data	Percentage of programmes with 30% Families with Challenges and 70% Families without Challenges
Dose received	N/A	Fidelity assessment sheets	Percentage of activities reporting interest of: young people; and parents/carers as 3/4 (on scale of 1 [low] to 4 [high])
Reach	N/A	Co-ordinator data	Percentage of families allocated to intervention, attending ≥5 sessions without missing 2 sessions in a row
			
Inputs	SFP 10-14 UK approach - free childcare, travel, refreshments	Fidelity assessment sheets	Percentage of sheets with positive evaluation of quality of childcare, refreshments and travel arrangements
	Venue	Fidelity assessment sheets	Percentage of sheets with positive evaluation of accommodation quality
	Materials and equipment	Fidelity assessment sheets	Percentage of sheets with positive evaluation of materials/equipment

a Kumpfer, Molgaard and Spoth (1996) indicate 5 as the minimum number of families per programme; although the aim was for each group to recruit 10–12 families, no minimum (below which the programme could not run) was specified for Project SFP Cymru delivery teams over and above the guidance provided by the developers of the original US-based version of SFP 10-14.

#### 2.5.1 Routine data from co-ordinators

SFP 10-14 co-ordinators were responsible for collecting information on dose delivered (programme/session dates), intervention reach and retention (family attendance), family characteristics, and venue type. For 33 of the 56 programmes they were also asked to collate information on staffing numbers and consistency. Data was passed to the research team for analysis.

#### 2.5.2 Fidelity assessment

To assess adherence to planned programme content during SFP 10–14 sessions, facilitators completed a fidelity assessment sheet for each programme hour. Data were collected for 50 of the 56 programmes delivered. Based on the 33 sessions for which staffing data was available the response rate (percentage of total possible assessment sheets) was 48%. The fidelity assessment sheet was based on schedules provided by Leland Molgaard, SFP 10–14 trainer in Iowa, and used in previous evaluations of SFP 10-14 in the USA (e.g. Byrne et al*.*, 2010). It comprised ordinal scales on coverage of activities, timing, and dose received (reception by participants); and free text responses on the quality of intended inputs: accommodation, equipment/materials, refreshments, childcare and transport. These responses were coded as positive, neutral or negative. To assess reliability of facilitator reports a sample of 47 sessions from the 50 programmes for which data were available were observed and scored by researcher observers using the same fidelity assessment sheet. Two observers attended sessions 2, 4, 5 and 6, either singly or together, covering all counties. These sessions were selected in order to encompass different stages of each 7 week group, and the various topic areas/activities. When two observers attended, both assessed the same two hours, i.e. the family hour and the young people's or parents/carers’ hour, so that they could compare scores. Differences in scoring were discussed and resolved.

#### 2.5.3 Semi-structured interviews

Semi-structured interviews with SFP 10–14 trainers (n=2), programme co-ordinators (n=9), coordinators’ managers (n=7), and facilitators (practitioners from local agencies who undertook training to deliver programme sessions) (n=20) (Table 2) explored context and systems for implementation; and acceptability to families and staff. All staff in post during the process evaluation were invited to participate. All but three interviews were audio-recorded (with participants’ permission). Eleven facilitators participated in one-to-one interviews. Nine took part in group/joint interviews.

**Table 2:** Numbers and percentages of SFP 10–14 implementation staff participating in interviews.

**Source**	**Total population (at point of recruitment)**	**Invited** (n)	**Number participating**	**Number participating (% total)**	**Number participating (% invited)**
Trainers	2	2	2	100	100
Co-ordinators (1^st^/only interviews)	13	10	9	69	90
Co-ordinators (2nd interviews)	2	2	2	100	100
Managers	9	7	7	78	100
Facilitators	203	203	20	10	100

### 2.6 Analysis

#### 2.6.1 Quantitative data

Descriptive statistics were calculated for variables representing adherence to programme content: numbers of staff and participants; participant engagement and attendance; and quality of accommodation, childcare, transport, refreshments, and materials/equipment. Agreement between observers was assessed using intra-class correlation coefficients (ICC). Agreement between observers and facilitators on adherence was assessed by calculating the percentage of agreement. Kappa statistics were also considered. However, Kappa scores were low due to extreme distributions of the marginal totals (facilitators only rarely rated activities as not/hardly/partly covered) (Feinstein & Cicchetti, 1990), and so did not provide an accurate representation of agreement.

#### 2.6.2 Qualitative data

Thematic content analysis (Braun & Clarke, 2006) identified themes which were developed into an analytic framework, based on the process evaluation aims, interview guides, and additional themes identified during the analysis process. Atlas.ti 6 software (ATLAS.ti Scientific Software Development GmbH, Berlin, Germany) was used to code data from interview transcripts so that themes could be related to different participant groups and implementation counties. The coding framework was refined through double-coding of seven interviews.

#### 2.6.3 Analysis plan

**Fig. 2:**
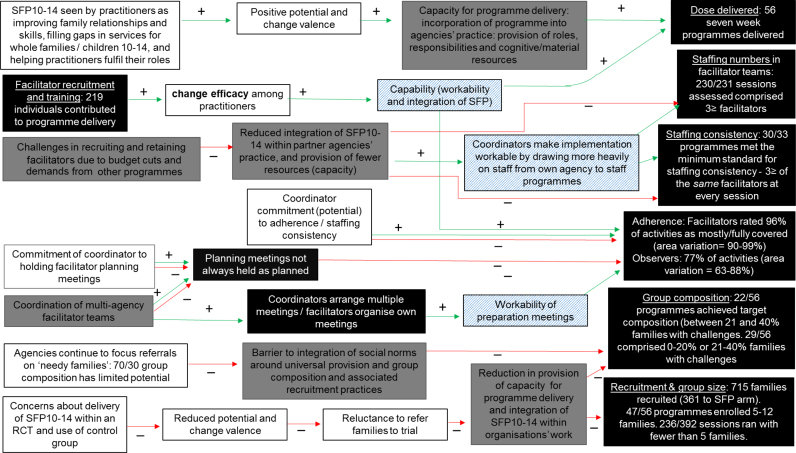
: Summary of main results: Implementation of intended programme inputs/activities using ENPT as an organising framework to understand key influences. ENPT components: potential (white boxes); capacity (grey boxes); capability (patterned boxes); contribution (black boxes). Processes aligned with SFP 10–14 logic model (Fig. 1) are shown in green (+). Those which may reduce/disrupt alignment are shown in red (−). Underlined terms are planned inputs/programme activities in Fig. 1. (For interpretation of the references to color in this figure legend, the reader is referred to the web version of this article.)

Similar to the approach of McEvoy et al. (2014), qualitative data coded to each implementation-related theme was read against the core constructs of ENPT (*capability*, *capacity*, *potential* and *contribution*). An initial definition of each ENPT construct – with some terms adapted for the study setting, was produced - a process previously found to be useful in applying a theoretical framework to existing thematically coded data (MacFarlane & O’Reilly de Brun, 2012). Two researchers then independently coded each theme using the agreed ENPT construct definitions (which were revised where necessary as analysis progressed). Disagreements in coding were resolved by the two researchers, with input from a third member of the team in a small number of cases. The completed analysis was then reviewed by the wider process evaluation team (by reading the original summaries of thematic codes and the subsequent coding of the data against ENPT constructs). They assessed there to be a good fit between the data in the implementation-related codes and key ENPT constructs. Following this stage, we built a model (Fig. 2) to map how aspects of practitioner agency (*capability*) and the delivery system (*capacity* and *potential*) shaped the extent to which programme inputs and activities were delivered as intended (*contribution*) - described in the quantitative data.

## 3 Results

### 3.1 Implementation of programme activities (*contribution*)

715 families were enrolled in the RCT; 361 were allocated to receive SFP 10–14. Eighty-four percent (47/56) of programmes enrolled 5–12 families. However, not all families attended every session and 60% (236/392) of sessions ran with fewer than 5 families. Eighty percent (287/361) of families attended at least one session. Sixty percent (218/361) were classed as having received the intervention – defined as attending five or more sessions without missing more than one session in a row, and 33% (119/361) attended all seven weeks. There was no evidence that smaller groups reduced participant engagement – which was rated as high in 94% of 22, 407 facilitator ratings. Table 3 shows the composition of the groups according to families with/without challenges. Thirty-nine percent (22/56) of programmes achieved the target composition (groups with 21- 40% families with challenges), with most of the remainder (29/56) comprising 0–20% or 41–60% families with challenges. Five programmes included >60% families with challenges.

**Table 3:** Distribution of group size for the trial as a whole and the varying group compositions achieved.

**Group composition and programme size**		**Number of families enrolled (group size)**						
		**≤4**	**5**	**6**	**7**	**8**	**≥9**	**Overall**
**Group composition**	**0 -20% FWC**	1	2	4	6	0	1	14
	**21–40% FWC**	4	4	3	4	5	2	22
	**41–60% FWC**	2	2	2	4	2	3	15
	**>60% FWC**	2	0	1	1	0	1	5
	**Overall**	9	8	10	15	7	7	56

A total of 219 facilitators were involved in delivering 56 programmes. Of these, 180 (82%) were female and 96 (44%) were practitioners working with children/families, employed by third-sector agencies or local authority services. Smaller numbers worked in adult social care (12), health (3), education (29) or the emergency services (10). The remainder were SFP 10–14 co-ordinators (11), managers (8), administrators (11), students (10), volunteers (17), or data were missing (12). Only one session ran with fewer than 3 facilitators (the recommended minimum). The number of different facilitators involved in the running of single programmes ranged from 4–10, and 30 programmes (91%) met the minimum standard for staffing consistency - three or more of the *same* facilitators at every session.

The intraclass correlation coefficient (ICC) of adherence scores from the two observers was 0.73 (95% CI 0.65–0.79), representing good agreement (Cicchetti, 1994). Overall, facilitators and observers agreed with each other 83% of the time (between-county range 73–93%). 96% of 13675 facilitator ratings scored activities as mostly/fully covered. This varied across counties from 90% to 99%. Three hundred and fifty three observer ratings scored activities as mostly/fully covered (77% of observer ratings; between-county range 63–88%) (Table 4).

**Table 4:** Coverage of SFP 10–14 activities: reports from facilitators, observers and observed agreement.

**County**	**% activities mostly/fully covered**		**% observed agreement (n=760 paired facilitator / observer activity ratings)**
	**Facilitators (n=13675 activity ratings)**	**Observers (n=456 activity ratings)**	
A	94	63	78
B	97	88	93
C	99	80	85
D	90	83	87
E	98	80	81
F	96	70	73
G	99	79	80
Overall	96	77	83

### 3.2 Project SFP Cymru set up: positive potential and high capability

At the beginning of the study *potential* was positive for the intervention across all sites. SFP 10–14 was welcomed as filling a gap – by serving children aged 10–14, and providing support for both parents/carers and young people. Eleven interviewees in four counties (5 coordinators, 3 managers, 3 facilitators) described being keen to work with parents/carers and children together. The belief that SFP 10-14 benefited families by improving relationships and providing family members with skills for dealing with difficult situations was mentioned in five counties (2 coordinators, 3 managers, 4 facilitators). Interviewees saw that SFP 10–14 worked in a way which would help them in their professional support roles:

*… the big thing that appealed was that the families have come together. Our parenting group was always just the parents and we did run a teenage group but we never did anything alongside each other. [ …] So when we heard about Strengthening Families we wanted to know more and see how we could get involved with being part of it really*. (Facilitator).

Positive *potential* energised local partnerships to create *capacity* for SFP 10–14, by incorporating the programme into everyday practice through rearranging roles, responsibilities and resources. SFP 10–14 co-ordinators were provided with office accommodation and administrative assistance; staff from local agencies were released for training as SFP 10–14 facilitators. Some practitioners were strongly motivated to provide direct support (e.g. working as a facilitator) and indirect support (e.g. referring families to the programme) and four had done the training and/or programme sessions in their own time.

### 3.3 Positive potential, high capability and decreasing capacity: Factors influencing implementation of planned programme activities

Nineteen interviewees (12 facilitators, 2 managers, 5 coordinators) said they had enjoyed the facilitator training. Eight (6 facilitators, 1 manager, 1 coordinator) reported valuing the facilitator manual as a practical guide because it was detailed and easy to follow. According to one trainer, training tended to increase change efficacy related to programme content because it dealt with objections or misunderstandings about SFP 10–14, so that trainees could gain an understanding of programme theory and design. Practitioners therefore understood how SFP 10–14 worked as a whole. One facilitator commented that:

*[…] you really saw how the activities were mirrored for the young people and the parents, there was a different approach with the DVD for the parents and the games for the young people, you could see how they were working towards a common aim and how that was embedded then in the family session.*

Training guidance was that a meeting should be held the day after each SFP 10–14 session to allow facilitators to debrief and prepare for the next session. These meetings included agreeing roles and responsibilities, and addressing issues relating to group dynamics and participants’ needs, to ensure that programme activities were covered as intended. However, three interviews with facilitators in County D indicated a link between coverage rates and staff preparation; they said they had needed to improvise during SFP 10–14 sessions because there had been no preparation meetings and they did not know what to do:

*We didn’t kind of decide until we got there on the session what - whether it would be parents or whether it would be this or that or the other. So it was - did feel a little kind of off the deep end cos the training had been probably a good six months before.* (Facilitator).

Lower coverage rates were found in counties where regular meetings were not held or did not fulfil intended functions. In County F, with the second-lowest observer-rated coverage and least observer-facilitator agreement, meetings lasted 30 minutes, with little time for discussion: *“[…] it was just purely a ‘sit down, this is what you’ll be doing’* (Facilitator). In County B, with the highest observer-rated coverage and agreement rates, interviewees consistently reported that meetings were held before each programme session. Meetings lasted two hours, which facilitators felt was the minimum required for them to debrief, plan, practise and get to know each other.

Several practitioners (4 coordinators, 4 facilitators) described how meetings were difficult to arrange where facilitators were working for different organisations in separate locations and coping with work demands which were too urgent, heavy or inflexible to allow them to attend every week. When facilitators were unable to attend meetings, co-ordinators in Counties A, B, C, E and F contacted them individually. In County A, with the lowest observer-rated coverage, facilitators were frequently unavailable for meetings and the co-ordinator said that during one programme *“four planning meetings instead of one”* were held every week. However, one-to-one contact would not have been a complete substitute for staff meetings, which were described in eleven interviews (2 coordinators, one manager, and 8 facilitator interviews across 6 counties) as important to build relationships, discuss issues arising with participants, support team working, get other facilitators’ perspectives on what had happened during a session, and build inexperienced facilitators’ confidence.

Difficulties in assembling facilitators for meetings were part of more general problems affecting facilitator recruitment. Co-ordinators in five counties said they struggled to recruit and retain facilitators from local agencies. *Capacity* to release staff decreased following cuts in public-sector jobs and funding: “*It's impossible [to get facilitators]. Especially now […] where funding has been cut and people are tending to have to job share and won’t be released from their role and things like that, so that's really difficult.*” (Coordinator) Seven practitioners described how facilitators had been obliged to change, or had lost, their jobs. Three managers and a trainer explained how the resulting reduction in the pool of facilitators was aggravated in some counties by their small geographical size or competition from other programmes:

*[…] SFP isn’t the only programme in [name of area] that's using facilitators working within their core role. We have the [name] programme which also requires there to be facilitators and we have the [name] programme which is an emotional and mental health programme for children and young people and again that'*s *being run through volunteer facilitators, people that have been a volunteer from their service, and because there's a limited pool of workers that the facilitators can come from and with budget cuts people being - people's work load going up it actually makes it more difficult, the pool of potential facilitators gets saturated very quickly and I think we would struggle if we were to try and recruit many more to be honest. (Manager)*.

These difficulties suggested that fidelity to staffing standards would be low but quantitative findings indicated the opposite. Further analysis of interview and routine data revealed that co-ordinators in counties A, E and F had relied increasingly on colleagues in their own organisations to staff SFP 10–14. This circumvented barriers to recruiting staff from multiple outside agencies and rendered achievement of staffing standards workable. The programme had become embedded within coordinators’ employing organisations, who had made a commitment to lead implementation and make organisational changes (e.g. staff recruitment, provision of accommodation) necessary to fulfil this role. This meant that they were more able and willing to sustain an increasing burden of staffing compared with partner agencies where the programme was less embedded, and who could stop releasing staff without impacting on the delivery of their core work. Achieving multi-agency staffing was challenging in three counties, but appeared to have been maintained in counties B and D. Co-ordinators in both counties were in post throughout the implementation period, which may have increased workability of multi-agency staffing and enabled them to sustain relationships with professionals in other agencies. All other co-ordinators were in post for shorter periods.

High staff turnover did not appear to be a risk to other aspects of implementation, and evidence from county D suggested that co-ordinators’ time in post was less important than their individual *potential*. The County D co-ordinator lacked commitment to strict fidelity, did not think it was important to have the same facilitators at every SFP 10–14 session and reported changing programme content to make it more interesting. This negative *potential* may explain why preparation meetings were rarely held, and why even with reduced *capacity*, multi-agency staffing would have been more easily achieved if managers were not being asked to release the same staff for seven consecutive SFP 10–14 sessions and meetings. Some County D facilitators contacted each other independently to prepare for sessions (reported by four facilitators), which would have attenuated the co-ordinator's negative influence on coverage rates.

Delivery of supporting inputs for the programme was generally good. Facilitators’ feedback and routine data from co-ordinators indicated that attendance at SFP 10–14 sessions was facilitated in accordance with the SFP 10–14 approach. Most comments on childcare, refreshments, travel arrangements, accommodation, materials and equipment were positive, ranging from 60% of comments on materials to 81% of comments on childcare.

### 3.4 Decreasing potential, capability and capacity: Barriers to trial recruitment

All co-ordinators reported difficulties in recruiting families. Two co-ordinators explained that they were seconded from organisations providing support services to vulnerable and needy groups and their professional contacts were with agencies primarily concerned with such families. Identifying families to take part in a universal programme presented a challenge because co-ordinators were recruiting from the general population rather than a clearly identified client group which was the norm. Three coordinators and two managers felt that agencies did not fully understand that SFP 10–14 was universal, and continued their normal practice of referring only “needy” families. Co-ordinators’ and facilitators’ organisational affiliations also fed a more general perception that SFP 10–14 was for families with expressed needs or problems.

As recruitment proceeded, practitioners in some agencies perceived a divergence between their goals of supporting individual clients by offering them SFP 10-14 and provision of the programme in the context of an RCT which restricted access to families willing to participate in the trial, and who were randomised to the intervention arm. Managers (n=2), coordinators (n=3) and facilitators (one group interview) indicated that some professionals became reluctant to refer families for these reasons:

*[…] when people were hearing that ‘oh xyz they’d all been referred, they’re in the control group, next one's in the control group, next one'*s *in the control group’, people were starting to […] say ‘What's the point in me referring?’ (Manager)*.

Thus, positive change valence was affected by the research context which reduced integration of the intervention within local systems. The impact of recruitment difficulties was felt at trial, programme and session levels. Implementation was extended for 12 months so that enough families could be recruited to provide the required sample size for the trial.

### 3.5 Sustaining potential and capacity beyond the end of the trial

Decisions on long term funding at national (and to some extent county) level were dependent on the findings from the RCT. This not withstanding, the value which staff placed on SFP 10–14 drove high levels of *potential* among practitioners towards sustaining implementation of the intervention beyond the period funded by the randomised controlled trial. Staff in six counties described plans for implementing the programme beyond the trial funded period:

*We are going to try and find the money if we can. We wouldn’t want it to go out of our portfolio of services. Strengthening Families is something that we feel we are getting good feedback from families [on]. Families want to engage with it, and it'*s *filling a gap for us in services that we have for teenagers and so definitely we would want to keep the project and take it forward further.*

*(Manager)*

*I’m very glad we’ve got the programme, I’d like to keep it, I’ve already been talking about how we might be able to sustain it beyond the life of the trial. It's very popular, parents who come on it love it, the facilitators are so enthusiastic about the programme, it just makes sense to everybody and they are very keen to continue delivering. So there's a lot of good will around the programme and beyond the life of the trial we will do everything we can to try and keep it going and not lose that expertise and that opportunity*.

*(Manager)*.

One county had already received some follow-on funding. Securing dedicated funding from local commissioners was seen as important but was recognised to be challenging within a context of budget cuts. In some cases, staff described how they were thinking strategically about those potential funders whose aims or work SFP 10–14 was aligned with or could help meet:

*[We are] looking at different initiatives that are coming out, like Team Around the Family which has just started with us, how you link into that? Because almost all of their desired outcomes are covered by Strengthening Families. I’m sitting there ticking them off in my head.*

*(Coordinator)*.

County C aimed to secure dedicated funding for an SFP 10–14 coordinator, either through a ‘standalone’ grant, or by integrating the programme within their portfolio of services and allocating resources to its implementation. In Counties A and B the intention was to employ a coordinator who would be responsible for the delivery of multiple programmes, including SFP 10–14, which would help sustainability but might reduce the number of programmes that could be delivered each year. Alongside financial resources (e.g. for employment of a coordinator) another challenge identified in most counties was the need to maintain and coordinate a network of facilitators from multi-agency partnerships who could staff programmes. There was uncertainty as to whether agencies would continue to provide time off in lieu to staff who worked as facilitators on the programme outside of normal working hours.

Many interviewees believed that linking the programme more closely with schools could increase *capacity*, by creating semi-autonomous delivery teams, providing access to suitable accommodation free of charge, and facilitating recruitment of families from the general population. Other potential solutions to the challenge of coordinating delivery teams were put forward, including uni-agency delivery, and identifying a dedicated facilitator from each partner agency, so that coordinators were working mainly with individuals who had significant prior experience of delivering the programme, and thus required less support.

In general participants indicated that the programme was likely to continue to be delivered in its intended form. However, in County A it was suggested that parts of the intervention (i.e. week sessions) could be delivered in standalone form, such as the work on making good choices, which might be offered to schools whose pupils were selecting subjects. Although many practitioners remained committed to the 70/30 model (as they recognised the difficulties of delivering the intervention to groups comprised solely of families with challenges), there was a recognition that partner agencies would be likely to focus referrals on families with challenges, and that once the programme was not part of an RCT, referral rates would increase as objections to randomisation were no longer an issue.

## 4 Discussion

In this paper we have drawn on Extended Normalisation Process Theory to evaluate the delivery of a complex intervention – the Strengthening Families Programme 10–14. We have used the theory as a framework to understand how the interplay between the intervention and local delivery systems shaped implementation. This was achieved by considering how practitioner agency (*capability* and *contribution*) and delivery systems (*capacity* and *potential*) interacted with the intervention (May, 2013), the extent to which it was delivered as intended, and how this varied over space and time. The findings provide valuable contextual information for the RCT which is assessing the effectiveness of SFP 10–14 on behavioural outcomes, and important insights into the extent to which the programme can be delivered as intended within a UK setting, following adaptation from the original US version.

Overall SFP 10–14 was delivered with good fidelity, and families received the intended intervention. Adherence to programme content by facilitators was high, but with some variation across delivery sites. These findings mirror those of previous evaluations of SFP 10–14 in the USA (Cantu et al., 2010; Spoth, Guyll, Trudeau & Goldberg-Lillehoj, 2002), and provide evidence that its implementation functions in broadly similar ways across contrasting national contexts, with key activities which are hypothesized to produce behaviour change being delivered in line with the programme's logic model.

In our study, high *potential* among practitioners towards SFP 10–14 appeared to have a positive influence on implementation. Practitioners valued the intervention (and were committed to delivering it) because they believed SFP 10–14 could help families and fill gaps in existing services. Through facilitator training, delivery staff gained an understanding of what they were required to deliver and how programme components were intended to work and interact, thus enhancing their *capability* to deliver it. Variation in adherence to planned programme content appeared to be influenced by levels of *capacity* to achieve coordination across multi-agency networks, though coordinator *potential* was also important. In particular, the organisation and quality of facilitators’ preparatory meetings – which were designed to optimise delivery processes and group dynamics - varied significantly. Programme adherence appeared to be higher in counties where the meetings were held regularly and fulfilled their intended purpose. Nonetheless, it is possible that the quality of meeting arrangements was associated with other aspects of implementation which also affected adherence. Targets for staffing numbers and consistency were met, but involvement of facilitators from multi-agency networks became increasingly difficult as levels of *capacity* reduced – partly due to cuts in public-sector jobs and funding. However, coordinators were sometimes able to overcome these challenges, for example by drawing more on staff from the programme's host agency or organising multiple meetings.

Family recruitment into the trial was challenging, and group size and composition targets were not always achieved. Practitioners’ concerns about random allocation of participants to intervention/control arms, and universal provision of the intervention (as opposed to focusing on families with support needs) impacted levels of *potential* and their willingness to refer families. However short-term funding, job losses in partner agencies, and competition from other programmes also played a part in reducing *capacity* to support recruitment – a situation many interventions face.

Despite practitioners’ concerns about the use of an RCT design, some aspects of the trial may have had a positive impact on implementation *capacity* and *capability*, particularly comprehensive funding for staffing, multi-agency practitioner training, and resources to enable family attendance – levels of funding which were not always sustained after the end of the study. Good rates of fidelity could also have been shaped by the heightened levels of monitoring which the trial introduced, such as observation of programme sessions, reinforcing practitioner commitment and enhancing intervention *contribution*.

Given the aim of forming groups to comprise a specific ratio of families with/without challenges to optimise group functioning and dynamics, the difficulties in consistently achieving the specified composition were theorised to be important for intervention *contribution* and implementation fidelity. However, although groups did not always achieve the target, most programmes comprised a mix of families with and without challenges in which the latter formed the majority. Only 5 groups comprised more than 60% families with challenges. The generally high levels of adherence and engagement achieved suggest that deviations in group size/composition from the intended formations may not have been significant enough to impact negatively on group dynamics or the delivery of intervention activities.

Our study – the first to our knowledge to use ENPT as the analytical framework for an empirical study of a social intervention, has several strengths. It builds on previous process evaluations of SFP 10–14 (Byrne et al., 2010, Spoth et al., 2007, Spoth et al., 2002) – and other trials of parenting/family interventions, which have focused primarily on assessment of pre-specified structural aspects of implementation (Bisset et al., 2009, Hawe et al., 2004), by theorising implementation processes in order to provide greater understanding of how and why the programme was delivered as it was. This was done by integrating quantitative assessment of implementation (e.g. adherence, recruitment) and qualitative investigation of practitioners’ agency and the dynamics of local delivery systems, using ENPT as a theoretical framework. The qualitative dataset is important because it aids interpretation of implementation and programme behavioural outcomes (Moore et al., 2014), and offers insights into the kinds of conditions needed for the intervention to be delivered as intended when transferred to new settings. ENPT provided an effective framework to examine how intervention implementation and its variation may be shaped by interactions with local delivery systems and practitioner agency. Through using it we have addressed calls to pay greater attention to the theorisation of implementation processes and the role of intervention-context interactions in shaping their variation (Bisset et al., 2009, Bonell et al., 2012, Glasgow et al., 2006, Hawe et al., 2004), which can help optimise the explanatory value of process evaluations.

Although other implementation frameworks assess moderators of fidelity (e.g. Carroll et al., 2007) and examine intervention adoption and maintenance (e.g. Glasgow et al., 1999), ENPT's distinctive contribution is that it offers a theory to help understand implementation processes, and how and why interventions are adopted and maintained (or not) over time. However, ENPT's focus is on how new forms of practice are embedded and integrated – it is not primarily a framework for assessing and explaining implementation fidelity – the main aim of process evaluations such as ours. We therefore used Linnan and Steckler's (2002) framework to identify those aspects of implementation that needed to be assessed, with ENPT employed to theorise implementation processes (identified in qualitative data) and the extent to which programme inputs and activities were delivered as intended (measured by quantitative data).

ENPT places considerable emphasis on the notion of implementation as an expression of agency. However, the agents in question appear to be mainly conceptualised as professional practitioners (e.g. nurses), rather than the participants who receive interventions. There is scope to consider further how the key constructs of ENPT can be applied to understand how participant (and non-participant) agency may shape whether interventions become integrated and embedded within delivery systems. For example, participants’ *potential* towards an intervention may influence levels of recruitment and the feasibility of long-term implementation. To achieve their hypothesized mechanisms, interventions such as SFP 10–14 require certain forms of *contribution* from participants, including participation in group activities, and the practising of skills within the home setting, which require cooperation and coordination between and across families (*capacity*).

We originally planned to conduct focus groups with parents/carers and children/young people who participated in the trial to explore the factors affecting participation in the trial and intervention, and families’ experiences of receiving SFP 10–14. However, despite significant efforts to recruit participants (including provision of free refreshments, organising transportation, and offering incentives), we were not able to recruit sufficient families to undertake the focus groups, and this is an important limitation of the study.

## 5 Conclusion

SFP 10–14 was delivered with good overall fidelity. Levels of adherence to programme content were high, though with some variation. Delivery of the intervention within the context of an RCT reduced levels of *potential* among some referrers, affecting the feasibility of planned recruitment and group composition targets. However, provision of material resources by the trial may have helped generate the *capacity* needed to make SFP 10–14 implementation workable, integrate the intervention within local delivery systems, and coordinate staff across multiple agencies. Trial-specific monitoring of implementation fidelity may have increased practitioner *potential* to deliver the intervention as intended, feeding through into intervention *contribution*.

Our findings will aid interpretation of outcomes from the effectiveness trial of SFP 10–14. Use of a pragmatic trial design (which aimed to assess delivery under ‘real world’ conditions) and analysis of the factors influencing implementation fidelity, provide valuable evidence about the extent to which our findings would be replicated when the intervention is used outside of a trial context, and the conditions necessary for successful delivery. Collection of data on implementation outcomes (e.g. recruitment, adherence, staffing levels, and group characteristics) would be valuable if the programme is delivered outside of a trial context– both to monitor what is delivered, and to develop understanding of how the programme operates in settings offering varying levels of *potential* and *capacity*.

Extended Normalisation Process Theory provided a useful framework to explain implementation processes, and their variation across time and space by examining interactions between the intervention and local delivery contexts. Our findings lend further weight to calls (Bisset et al., 2009, Moore et al., 2014) for process evaluations to examine both the structural and processual aspects of implementation - including interactions with local context, if they are to fully explain what is delivered and why.
